# Stress Responsive Proteins Are Actively Regulated during Rice (*Oryza sativa*) Embryogenesis as Indicated by Quantitative Proteomics Analysis

**DOI:** 10.1371/journal.pone.0074229

**Published:** 2013-09-18

**Authors:** Jin Zi, Jiyuan Zhang, Quanhui Wang, Baojin Zhou, Junyan Zhong, Chaoliang Zhang, Xuemei Qiu, Bo Wen, Shenyan Zhang, Xiqin Fu, Liang Lin, Siqi Liu

**Affiliations:** 1 Proteomics Division, BGI-Shenzhen, Shenzhen, China; 2 Beijing Institutes of Genomics, Chinese Academy of Sciences, Beijing, China; 3 College of Biological Sciences, China Agricultural University, Beijing, China; 4 Hunan Hybrid Rice Research Center, Changsha, China; University of Nottingham, United Kingdom

## Abstract

Embryogenesis is the initial step in a plant’s life, and the molecular changes that occur during embryonic development are largely unknown. To explore the relevant molecular events, we used the isobaric tags for relative and absolute quantification (iTRAQ) coupled with the shotgun proteomics technique (iTRAQ/Shotgun) to study the proteomic changes of rice embryos during embryogenesis. For the first time, a total of 2 165 unique proteins were identified in rice embryos, and the abundances of 867 proteins were actively changed based on the statistical evaluation of the quantitative MS/MS signals. The quantitative data were then confirmed using multiple reactions monitoring (MRM) and were also supported by our previous study based on two-dimensional gel electrophoresis (2 DE). Using the proteome at 6 days after pollination (DAP) as a reference, cluster analysis of these differential proteins throughout rice embryogenesis revealed that 25% were up-regulated and 75% were down-regulated. Gene Ontology (GO) analysis implicated that most of the up-regulated proteins were functionally categorized as stress responsive, mainly including heat shock-, lipid transfer-, and reactive oxygen species-related proteins. The stress-responsive proteins were thus postulated to play an important role during seed maturation.

## Introduction

Plant life begins as a zygote formed by the fusion of egg and sperm cells. After cell division and differentiation, the zygote will develop into a multicellular embryo containing all of the basic plant organs [1]. For a typical seed plant, the multicellular embryo will then experience severe dehydration and enter a dormant state during the later stages of embryogenesis. The morphologic changes during embryogenesis have been well studied and described, but the molecular changes that occur during embryonic development are still largely unknown [2]. Thus, discovering the proteins that respond to the different phases of embryogenesis would be helpful for understanding embryonic physiology and the mechanisms of dehydration tolerance in seeds.

Global changes in protein abundance have been well documented in several dicotyledonous plants and somatic embryogenesis. The seed development of the model legume Miyakogusa (*Lotus japonicas*) was studied by Dam et al. using two dimensional gel electrophoresis (2 DE) and gel-based liquid chromatography-mass spectrometry, resulting in 665 and 181 unique proteins identified for the seed-filling and desiccation stages of embryogenesis, respectively [3]. Proteomic changes during embryogenesis in soybean (*Glycine max [L.] Merr.*), castor (*Ricinus communis*) and rapeseed (*Brassica napus*) were also surveyed using 2 DE from 2 to 6 weeks after flowering [4]–[7]. Marsoni et al. applied 2 DE analysis to grape (*Vitis vinifera*) and discovered new proteins involved in somatic embryogenesis [8], and Pan et al. identified 24 differentially expressed proteins at five different points during somatic embryogenesis in the Valencia sweet orange (*Citrus sinensis Osbeck*), with 5 embryogenesis-related proteins being involved in glutathione metabolism and anti-oxidative stress [9]. These investigations also provided a hint that some stress-related proteins were pivotal in plant embryogenesis, such as heat shock proteins (HSP), peroxidases, glutathione S-transferases, and disease-resistance proteins [4]–[8] [10]. Early studies, however, were primarily focused on somatic embryonic development in dicots. In the field of monocot embryogenesis, there have been only two reports on rice: one discovering new putative roles for globulins [11] and the other identifying 275 differentially expressed proteins [12].

The proteomic studies of plant embryos reported so far have mainly used a gel-based approach, such as 2 DE. 2 DE is a powerful tool with the high-resolution detection of intact and modified proteins, but the protein detection upon 2 DE is biased towards hydrophilic [13] proteins with higher abundances [14] and intermediate *Mr* and pI properties. The technique is not sufficient for higher coverage and more accurate quantification in proteomic studies [15]. Thus, mass spectrometry-based quantitative approaches have recently emerged as important tools in proteomics studies [16]. Isobaric tags for relative and absolute quantification (iTRAQ) is one of the mass-based quantitative approaches and has become increasingly popular in the field of plant proteomics. For example, Owiti et al. adopted iTRAQ to identify approximately 4 000 cassava (*Manihot esculenta Crantz*) proteins [17], and Lan et al. quantified 2 882 *Arabidopsis thaliana* proteins with iTRAQ [18].

Multiple reactions monitoring (MRM) has been a principal tool for the quantification of small molecules in clinical chemistry for many decades. Because it contains two stages of mass filtering, the technique can result in high sensitivity, multiplexing capability, and precision for quantification. Recently, it has also been widely adopted for disease biomarker quantification studies [19]–[21], but it has rarely been used for plant protein quantification.

To study rice embryo proteins involved in embryogenesis, three factors were our primary concern regarding the technique. 1) How can the proteome coverage be improved? In this study, we determined that shotgun digestion with the peptides labeled with iTRAQ was the appropriate choice. 2) How can the iTRAQ results be confirmed by other experimental evidence? Rather than traditional methods such as western blotting, we employed multiple reactions monitoring (MRM), a more accurate quantification method, to verify the differential proteins determined by iTRAQ. 3) How are embryogenesis-dependent proteins defined? We did not adopt the generally used fold change as a threshold but instead used the Permutation Test with P<0.05 as the threshold to define significantly changed proteins. In this report, we identified 2 165 unique rice proteins (with 1% protein FDR from SCAFFOLD 3.6.5) including 867 differential proteins as embryogenesis-dependent. Of these, 40 embryogenesis-dependent proteins were further confirmed by MRM. Analysis of hierarchical clustering revealed two major abundance profiles during rice embryogenesis, with the protein abundance consecutively either up- or down-regulated. Furthermore, Gene Ontology (GO) classification demonstrated that the majority of the differential proteins belonged to the category of stress-related function. We have, for the first time, profiled the rice embryogenesis-dependent proteins with abundances that were sensitive to embryonic development. This dataset was reasoned to be valuable for exploring the mechanisms of rice embryogenesis and physiology.

## Materials and Methods

### Preparation of Rice Embryos

The rice strain 9311 (*Oryza sativa L. ssp. Indica*) was chosen for this study. The plants were grown in the spring of 2009 at the National Hybrid Rice R&D Center, Changsha, China (28°73′N, 11°37′E, altitude 40 m). Briefly, pre-germinated rice seeds were sown in March, 2009, and the seedlings were transplanted to a density of approximately 30 plants/m^2^. The field was flooded to a depth of 5 cm and highly irrigated throughout the study. Before planting, the soil was fertilized with 10 g/m^2^ of phosphorus and 30 g/m^2^ of potassium. Nitrogen from urea (7 g/m^2^) was supplied 10, 50, and 80 days after planting.

Spikelets were collected at five consecutive time points: 6, 12, 18, 24 and 30 DAP. For each time point, the spikelets were sampled at 5∶00 p.m. and immediately prepared for embryo preparation. The embryos were immersed in liquid nitrogen first and then transferred to −80 degree Celsius refrigerator for later use.

For each sample collection time, some embryos were chosen, and their lengths and weights were measured. To estimate embryonic vigor, 20 rice spikelets were sterilized with 10% NaClO for 20 min, and the embryos were isolated using sterilized forceps and placed on 1/4 Murashige and Skoog medium for germination. Four days after germination, the bud lengths, representative of embryonic vigor, were measured.

### Protein Extraction

Two biological replicates were prepared for each embryogenesis stage.

The first biological replicate was used for iTRAQ/Shotgun analysis. The rice embryos were ground to a fine powder and suspended in 0.5 M triethylammonium bicarbonate (TEAB) buffer with 1 mM phenylmethyl sulfonyl fluoride and 0.1% SDS, followed by sonication for 5 min and centrifugation at 20 000 g for 30 min. The supernatant was transferred to another tube, 0.5 M TEAB buffer was added to the pellet to repeat the protein extraction, and the sample was again centrifuged at 20 000 g for 30 min. Proteins in the combined supernatant were reduced (10 mM DTT, 56°C for 60 min), alkylated (55 mM iodoacetamide, room temperature for 45 min), precipitated by precooled acetone at −20°C for 30 min, and then centrifuged at 20 000 g for 30 min. The pellet was washed twice with acetone, and the final pellet was dissolved in 0.5 M TEAB buffer with 0.1% SDS, sonicated for 5 min, and centrifuged at 20 000 g for 30 min. The supernatant was used for liquid digestion, and the protein concentration was determined using the Bradford assay.

The second biological replicate was used for the MRM confirmation. Rice embryos were ground to a fine powder and suspended in lysis buffer containing 8 M Urea, 10 mM DTT, 1 mM phenylmethyl sulfonyl fluoride, 2 mM EDTA, and 20 mM Tris-HCl, pH 8.5. After centrifugation at 20 000 g for 30 min, the protein concentrations of the supernatant were determined using the Bradford assay.

### In Solution Digestion and iTRAQ Labeling

For the iTRAQ/Shotgun experiment, 4 µl of trypsin (0.5 µg/µl) was added to 120 µg of the protein solution for protein digestion at 37°C for 12 h. Then, the same amount of trypsin was added again, and the sample was digested for 4 more hours. The digests were dried in a Speedvac. Each precipitate was dissolved in 40 µl of 0.5 M TEAB and mixed with 60 µl of isopropanol. The protein digests obtained from 6, 12, 18, 24 and 30 DAP were labeled with iTRAQ reagent (AB SCIEX, Framingham, MA, USA) 113, 115, 117, 119, and 121, respectively. The labeling reaction was conducted for 2 h at room temperature, and then the five labeled peptides were pooled together.

For the MRM confirmation, 20 µg of the protein solution from each embryogenesis stage was diluted 4 times with 25 mM NH_4_CO_3_, digested with 1 µl of trypsin (0.5 µg/µl) and incubated overnight at 37°C. The digestion was stopped by adding TFA to a concentration of 0.1%.

### SCX and RP nanoLC-MS/MS Analysis of Labeled Peptide

The pooled peptides were dried in a Speedvac and dissolved in 1 µl of buffer A (10 mM KH_2_PO_4_ in 25% ACN at pH 2.8). After adjusting the pH to 3 with H_3_PO_4_, the sample was fractionated using strong cation-exchange chromatography (SCX) on an HPLC (Shimadzu, Kyoto, Japan) equipped with a silica-based SCX column (250 mm×4.6 mm, Phenomenex, Torrance, CA, USA). A total of 28 fractions were collected at a rate of 1 ml/min with a buffer B (10 mM KH_2_PO_4_ and 2 M KCL in 25% ACN, pH 2.8) gradient as following: 0% for 10 min, 0–6% for 2 min, 6–12% for 20 min, 12–20% for 3 min, 20–50% for 1 min, and 50% for 2 min. The fractions were desalted with a strata-X 33 µm PolyRevStage SPE (Phenomenex) following the manufacturer’s instructions and dried in a Speedvac. Then, 30 µl of 0.1% FA was added to each dried fraction tube, and 0.1 µl of the re-dissolved solution was spotted on the target well of an Anchor-chip plate for MALDI-TOF testing. After the MALDI-TOF (Bruker Daltonics, Germany) testing, the peptides in the tubes with few peaks were pooled, resulting in 10 pooled SCX-separated fractions.

Each SCX fraction was loaded 3 times on a Prominence Nano HPLC system (Shimadzu, Kyoto, Japan) mounted with a 10 cm reversed phase C18 column (ID 75 µm, 3 µm particles, 200 Å aperture, 15 cm) and separated over a 40 min acetonitrile gradient from 5 to 35% in 0.1% FA combined with a Q Exactive mass spectrometer (Thermo Fisher Scientific, MA, USA). The data were acquired using a data-dependent data acquisition mode in which, for each cycle, the 15 most abundant multiply charged peptides (2+ to 4+) with an m/z between 400 and 1800 were selected for MS/MS with the 15-s dynamic exclusion setting.

### Label-free MRM Confirmation of Embryogenesis Dependent Proteins

All of the MRM confirmation experiments were conducted using the same experimental condition reported by Zhang [22] with some modifications in Triple Quad™ 5500 LC/MS/MS (AB SCIEX). Briefly, a C18 analytical column was prepared in our lab (ID 75 µm, 3 µm particles, 200 Å aperture, 15 cm), and 2 µg of embryonic peptides was subjected to the Enhanced Mass Spectrum-Enhanced Product Ion (EMS-EPI) mode for identification. Then, the EMS-EPI data were searched with ProteinPilot (AB SCIEX). Transitions for all of the identified peptides were designed by importing the ProteinPilot results files to MRMPilot (AB SCIEX) with the following peptide selection criteria: 1) unique peptide for a protein, 2) no cysteine or methionine, and 3) no variable modification. Finally, the 60 designed peptides (20 proteins with 2 peptides and 20 proteins with 1 peptide) and 180 transitions (3 transitions per peptide) were adopted to survey the protein digests from each of the 5 embryogenesis stages using the MRM mode with three injection repeats.

### Data Analysis

**Figure 1:**
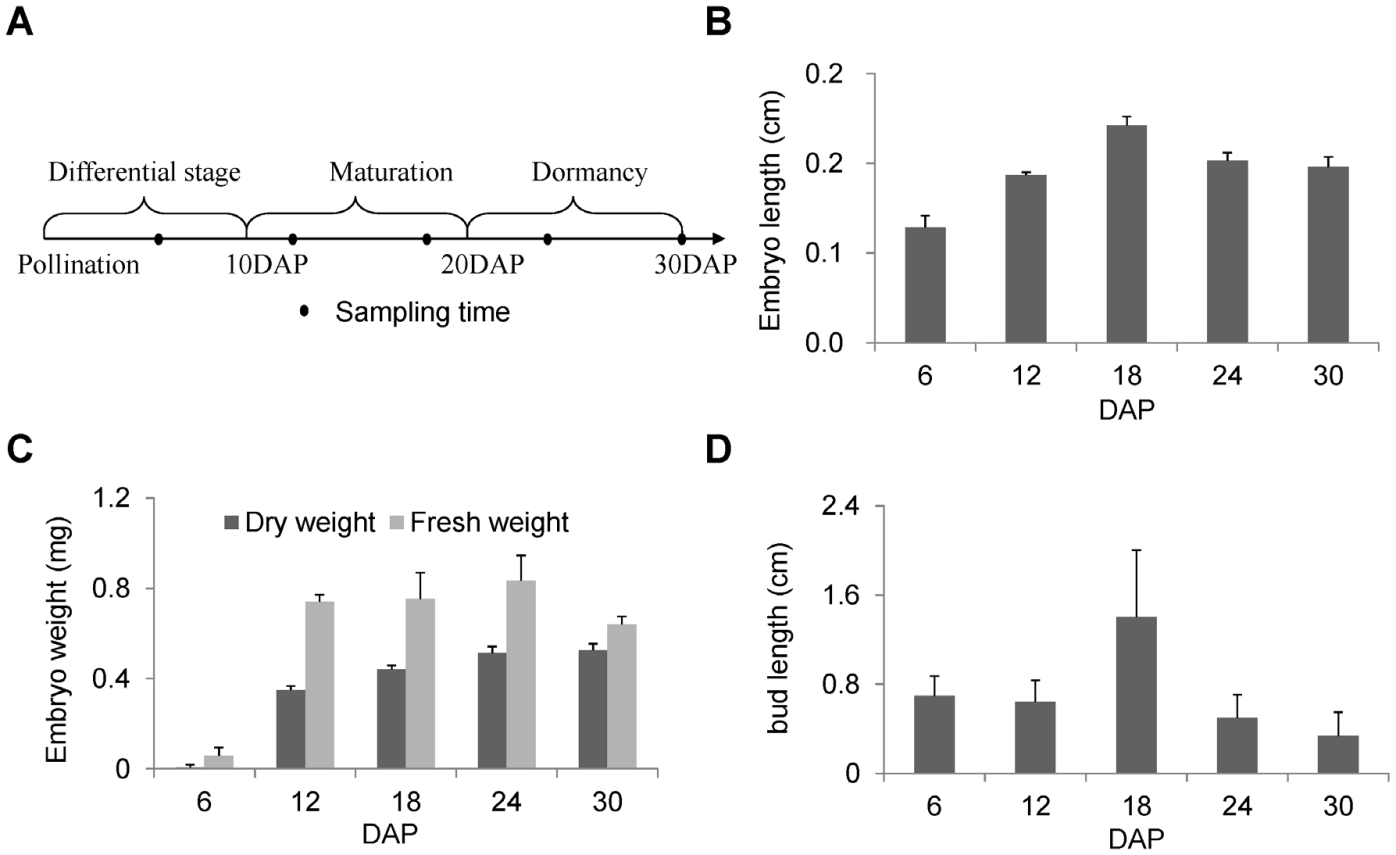
Rice embryogenesis stages and embryo phenotypes for each stage. (A) Embryogenesis stages and sampling time settings. (B) Pattern of embryo length change during embryogenesis. (C) Pattern of embryo weight change during embryogenesis. (D) Newly germinated bud length of embryos during embryogenesis. For (B) (C) and (D), the error bars indicate the standard derivation.

For the physical properties of the rice embryos, the average values of each parameter were statically calculated in the collected samples with the standard derivation calculated by Microsoft Excel, which was indicated as an error bar in Figure 1A.

For iTRAQ/Shotgun protein identification, the raw mass data were processed with Proteome Discover 1.3 (Thermo Fisher Scientific) and searched with in-house MASCOT software 2.3.02 (Matrix Science, London, U.K.) against the rice database 6.1 (67 393 sequences) of The Institute for Genomic Research. In the database search, full tryptic specificity was required with tolerance set at one missed cleavage. Carbamidomethylation of cysteine and iTRAQ 8-plex modification of the N terminus and K were set as fixed modifications. Gln->pyro-Glu of the N terminus, deamination of the N terminus, and oxidation of methionine were set as variable modifications. The initial precursor mass tolerance was set to 15 ppm, and the fragment ion level was set to 0.02 Da. The data files generated by MASCOT were processed using Scaffold Q+ (Proteome Software, Portland). For protein identification, FDR was calculated by Scaffold Q 3.6.5 using the algorithm published by Kall [23]. The expression data from Scaffold Q+ were further processed using the hierarchical cluster function of Gene Cluster software (Stanford, USA) under the default parameters without log transformation.

For the MRM confirmation, the raw data of the MRM was processed using MultiQuant 2.0.2 (AB SCIEX). After manual inspection and retention alignment, peaks with an S/N>10 were qualified for quantification.

To calculate the protein abundance of a protein in a specific embryogenesis stage, we used the sum of all the transition areas of a protein as an indicator of the protein abundance. The average areas of three technical replicates were used to indicate the final protein abundances of a stage. Finally, the abundances and ratios of each stage to 6 DAP were calculated by a perl programming script. The comparisons and scatter plots of the MRM and iTRAQ/Shotgun quantitative data were assembled using Excel (Microsoft Office 2010, USA).

## Results and Discussion

### Characteristics of Rice Embryos at Five Developmental Stages

Rice embryogenesis can be divided into three stages: cell differential stage (0–10 DAP), mature stage (10–20 DAP) and dormant stage (11–30 DAP) [24]. To monitor embryo protein changes spanning all of rice embryogenesis and the late embryogenesis stage in particular, rice embryos were sampled from five time points: 6 DAP in the cell differential stage, 12 and 18 DAP in the maturation stage, and 24 and 30 DAP in the dormant stage (Figure 1A). Then, the basic physical properties of these embryos were examined. During embryogenesis, the embryos grew steadily before the maturation stage and then shrank somewhat in the dormant stage (Figure 1B). The embryo fresh and dry weights both increased before 24 DAP. However, after 24 DAP, the dry weights remained at a constant level, whereas the fresh weights declined, which indicated that dehydration occurred in the later embryogenesis stages (Figure 1C). Embryo vigor was evaluated by measuring the bud lengths of germinated embryos. As depicted in Figure 1D, the bud lengths at maturation for stage 18 DAP were nearly two times longer than for the other four stages of earlier and later embryogenesis, indicating that embryo vigor reached the highest level at the maturation stage and was reduced at the dormant stage. The bell-shaped curve for the change in rice embryo vigor also indicated that the embryos were sampled during an actively changing period.

### Identification of the Rice Embryo Proteins

**Figure 2:**
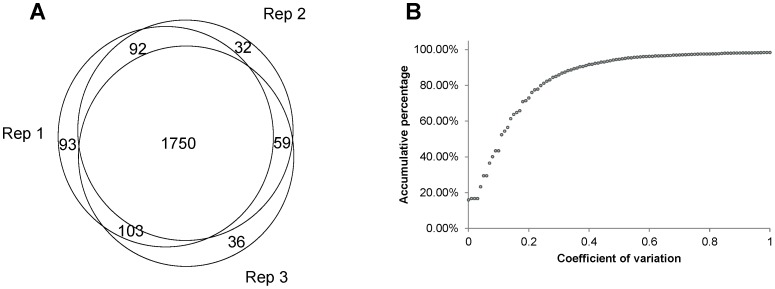
Evaluation of three technical replicates. (A) Venn chart showing the overlap of the identified proteins from the three replications. (B) Accumulated frequency graph of the coefficient of variation.

Using a Q Exactive MS with high resolution and fast MS/MS scan, we achieved satisfactory mass signals for rice protein identification and quantification. An average of 190 000 MS/MS spectra were acquired from each technical replicate, and the spectra annotation rates were approximately 11%. A total of 2 165 unique proteins were identified with 92% overlap (Figure 2A) among the three technical replicates, indicating that the data reproducibility in parallel experiments was acceptable for further proteomic analysis. The detailed information regarding the protein identification and the summarized information for each technical replicate are listed in Tables S1 and S2.

By using the GO identifier provided by the Institute for Genomic Research, we utilized WENGO (http://wego.genomics.org.cn) [25] to analyze the GO distribution of the identified embryo proteins (Figure S1 in five S1). In the molecular functional group, the identified proteins that work as metabolic enzymes, binding (protein binding and nucleotide binding) and transporters were ranked at the top of the category occupancy, suggesting that the relevant functions were important in rice embryos. Importantly, these observations were in agreement with those of our previous report [11]. In the biological process category, the proteins that participate in metabolism and the response to stimuli were at the top ratio in the identified embryo proteins, suggesting that embryogenesis is a process that requires proteins that protect against dehydration and provide the energy needed to maintain embryo vigor.

### Evaluation of the Quantitative Proteome Data

For accurate quantification, two criteria were established: 1) a protein for quantitative analysis must contain at least 2 unique peptides and 2) a peptide spectrum with all five reporter ions were accepted for quantification. A total of 1 744 proteins were qualified for iTRAQ quantification (Table S3). Of the triplicates, 90% of the quantitative data with coefficients of variation less than 40% reached the acceptable threshold in the quantitative proteomic studies (Figure 2B). Furthermore, we employed Scaffold Q 3.6.5 to estimate the differential proteins in response to the different rice embryogenesis stages. Calculated by using the iTRAQ data from the three technical replicates, the permutation test suggested that nearly 50% of the embryonic proteins (867/1744) showed significant abundance changes during embryo development (P<0.05, Table S3).

**Figure 3:**
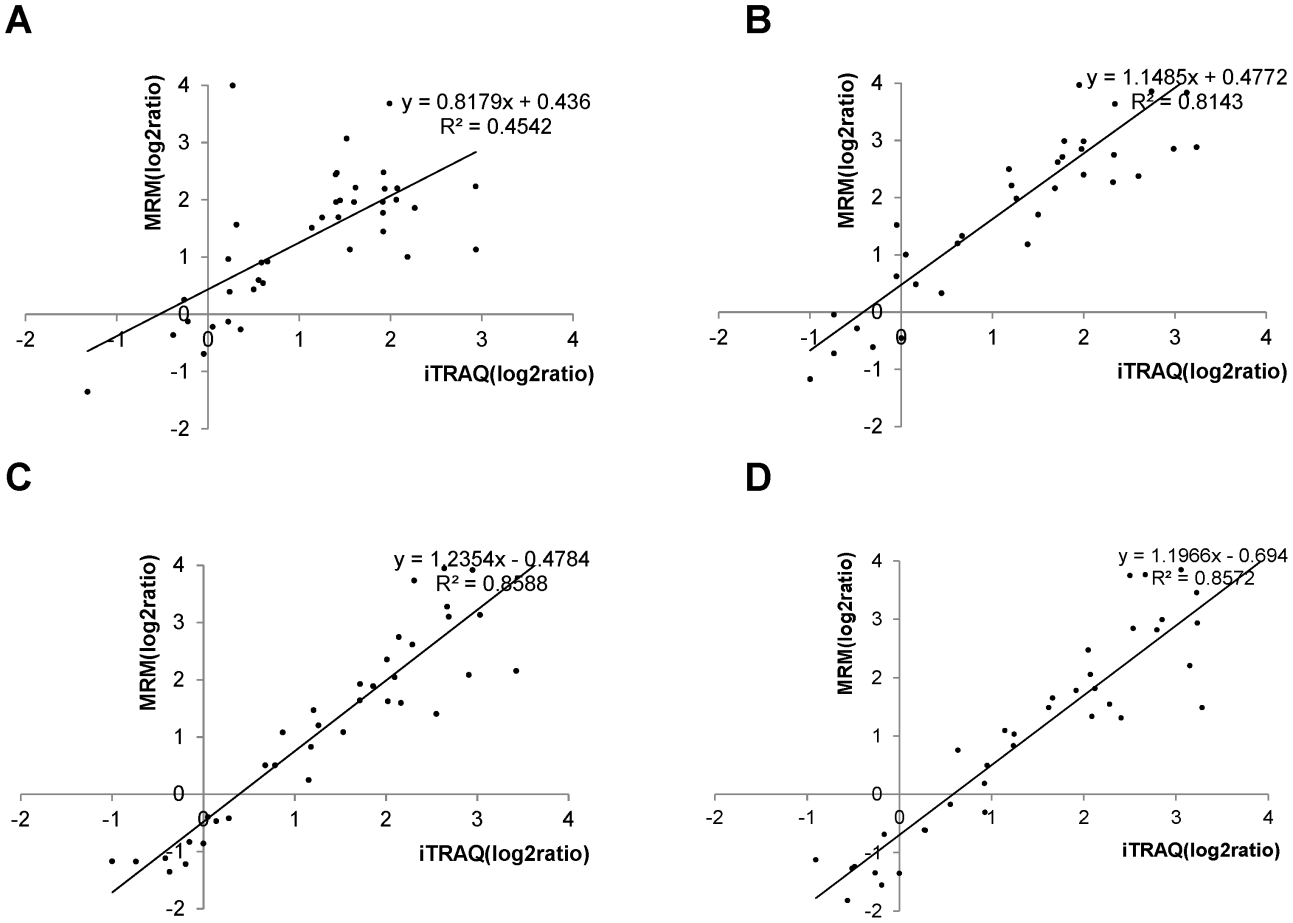
Scatter plot of iTRAQ quantified log2 (protein ratio) and MRM quantified log2 (protein ratio). (A) iTRAQ versus MRM log2(12 DAP/6 DAP). (B) iTRAQ versus MRM log2(18 DAP/6 DAP). (C) iTRAQ versus MRM log2(24 DAP/6 DAP). (D) iTRAQ versus MRM log2(30 DAP/6 DAP).

To confirm the quantitative data derived from iTRAQ, we adopted the EMS-EPI scan in Triple Quad™ 5500 LC/MS/MS (AB SCIEX) to seek the rice embryo proteins that produced strong MS/MS signals for the MRM assay. The scan survey revealed 63 rice proteins with acceptable MS/MS spectra, including 40 differential proteins found by iTRAQ/Shotgun. According to the MSMS information derived from EMS-EPI, we designed the corresponding transitions for these proteins and conducted label-free quantification of them using the MRM assay. As shown in Figure 3, the log ratios of the quantitative data from the MRM were basically correlated with those from iTRAQ with the regression coefficients greater than 0.8 except the quantitative correlation at the early embryogenesis stages (12 DAP). In our lab, 2 DE was adopted to analyze the rice proteome changes during embryogenesis [11]. In the early 2 DE study, 23 unique proteins were defined as embryogenesis-dependent, with 20 of these also identified and quantified as differential proteins by iTRAQ. The correlation curves of the abundance changes for these 20 differential proteins compared with the sampling time points are illustrated in Figure S2 in File S1. Approximately 75% (15/20) of the differential proteins shared between the two datasets showed similar time-dependent patterns in the abundance changes. Overall, the quantitative results based on iTRAQ labeling were strongly supported by the data derived from the other two quantitative technologies, MRM and 2 DE.

### Quantitative Profiling of the Rice Embryo Proteomes in Response to Embryogenesis

**Figure 4:**
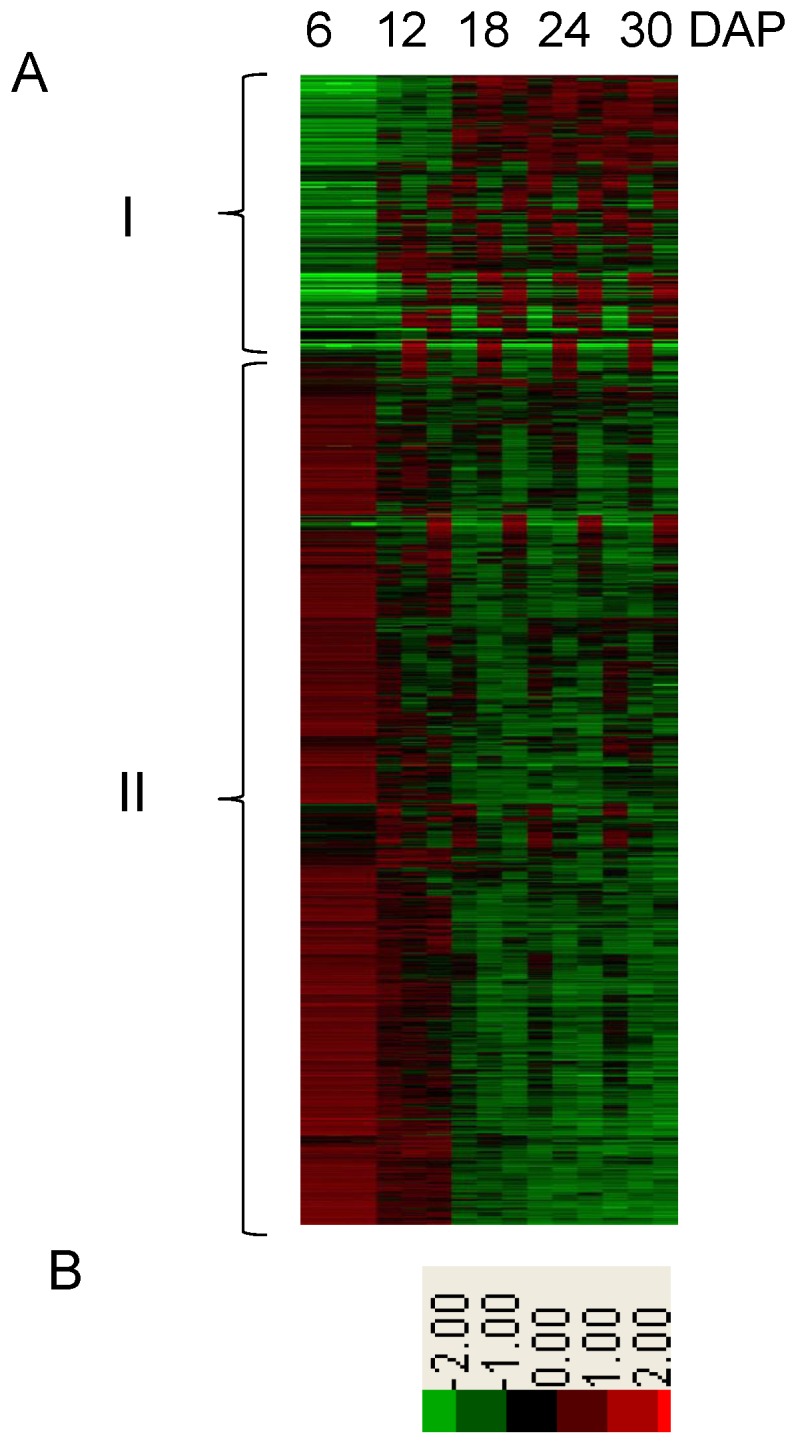
Cluster result of the significantly regulated proteins. (A) Cluster heatmap of significantly regulated proteins using the protein abundance information. The five columns from left to right are labeled 6, 12, 18, 24 and 30 DAP at the top of the heatmap. The left braces indicate the two groups classified by cluster analysis. (B) Color diagram of the heatmap.

Based on the entire set of quantitative data, we further characterized the functional features of the differential proteins. Taking the proteome at 6 DAP as a reference, combining the protein abundance data and the sampling time points, cluster analysis revealed that the differential proteins could be generally divided into two groups, consecutively up- (25%) and down-regulated (75%) in response to embryogenesis (Figure 4). The differential proteins were analyzed by GO categorization and broadly categorized into 16 molecular functions, 16 cellular compartments and 22 biological process groups (Figure S3, S4 and S5 in File S1). In the category of biological processes, the differential proteins involved in metabolism and stress-related processes were relatively dominant, similar to the result of the identified embryo proteins described in Figure S1 in File S1. As shown in Figure S5 in File S1, the proteins that responded to various stresses, such as abiotic, biotic and other stresses, occupied approximately 23% of all the up-regulated proteins. Moreover, of the differential proteins with stress-related functions, approximately 70% were up-regulated (Table S4). The evidence thus suggested that the stress-related proteins were important for rice embryo development.

### Stress-related Proteins were Mainly Up-regulated During Rice Embryogenesis

The plant disease response often mimics certain normal developmental processes. Tomáš et al. noticed that stress-related proteins could participate in all developmental processes [26]. Cooper et al. also reported that some rice genes were related to both stress defense and certain developmental processes [10]. With respect to embryo development, the best known stress-defense genes are the late embryogenesis abundant proteins, which are generally up-regulated during both embryogenesis and water-deficit stress [27]. Proteomic studies on plant embryogenesis have discovered many more proteins related to stress defense and embryo development. Heat shock proteins (HSP), glutathione S-transferase (GST), ascorbate peroxidase and copper/zinc superoxide dismutase have been demonstrated to be actively regulated during embryogenesis [4]–[6], [12], [28]. However, these studies offer little information about how stress-related proteins perform in the embryo due to the limited number of proteins identified. In this study, the higher coverage of the rice embryo proteome by the iTRAQ/Shotgun strategy offered a good opportunity to discover more stress-related proteins involved in embryogenesis.

#### Heat shock proteins (HSPs)

HSPs had been reported to be both up- and down-regulated during embryogenesis in Chinese fir (*Cunninghamia lanceolata* (Lamb.)) [29], maize (*Zea mays*) endosperm [30], *Vitis vinifera* [8], soybean [7] and rice [12]. A few HSPs in embryos and seeds were identified in these studies, most of which belonged to the higher molecular mass HSPs (hHSP, *Mr* >70 kDa). In contrast to early reports, our study identified more HSPs and multiple embryogenesis-dependent patterns for HSP abundance.

**Figure 5:**
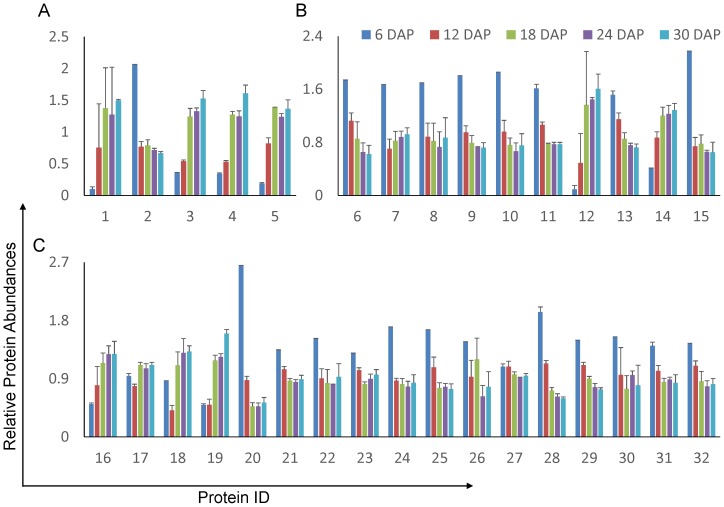
Relative abundance of heat shock proteins during embryogenesis. (A) HSP 20. (B) Higher molecular HSPs. (C) DnaK family HSPs. The error bars indicate the standard derivation. 1 indicates the HSP with Locus ID LOC_Os032870.1; 2, LOC_Os06g14240.1; 3, LOC_Os03g15960.1; 4, LOC_Os03g14180.1; 5, LOC_Os01g04370.1; 6, LOC_Os09g30412.1; 7, LOC_Os04g01740.1; 8, LOC_Os12g32986.1; 9, LOC_Os08g39140.1; 10, LOC_Os08g38086.3; 11, LOC_Os06g50300.1; 12, LOC_Os09g30418.1; 13, LOC_Os02g43020.1; 14, LOC_Os05g44340.1; 15, LOC_Os02g52150.2; 16, LOC_Os03g11910.1; 17, LOC_Os01g62290.1; 18, LOC_Os05g38530.1; 19, LOC_Os03g16920.1; 20, LOC_Os09g31486.1; 21, LOC_Os02g48110.1; 22, LOC_Os05g23740.1; 23, LOC_Os02g53420.1; 24, LOC_Os03g16860.1; 25, LOC_Os05g08840.1; 26, LOC_Os12g14070.1; 27, LOC_Os01g08560.1; 28, LOC_Os11g47760.1; 29, LOC_Os02g02410.1; 30, LOC_Os03g44620.2; 31, LOC_Os03g57340.1; 32, LOC_Os01g32870.1.

In total, 36 HSPs were identified in the rice embryos, and 32 were significantly regulated over the course of embryogenesis (P<0.05). The embryogenesis-dependent HSPs identified were broadly classified into three HSP families: HSP 20, hHSP and DnaK\J HSPs. For the HSP 20 class, 80% (4/5) increased in abundance during embryogenesis (Figure 5A). For the hHSP class, 75% (9/12) were down-regulated during rice embryogenesis. For the DnaK/J HSPs, 76% (13/17) were negatively regulated during embryo development. Briefly, we found that the abundance of most hHSPs decreased, whereas most small molecular HSPs (*Mr* <30 kDa) accumulated at late embryogenesis. These findings were in agreement with the observations made in *Cunninghamia lanceolata* (Lamb.), *Vitis vinifera* [8] and soybean [7].

This phenomenon implies that the three HSP families may have different functions during embryogenesis. HSPs can be used to stabilize protein conformation, prevent aggregation, and therefore maintain non-native proteins in a competent state for subsequent refolding in plants under stress. Thus, it is not surprising that so many HSPs were found to be regulated during embryogenesis. Our observations were supported by a number of investigations. HSP 70 was reported to potentially be involved in a repair function after desiccation rather than biochemical stabilization in the dry state for *Richtersius coronifer*, whereas HSP 101 was found to be a crucial element in thermal tolerance and thus highly induced in response to dehydration in Arabidopsis [31]. With regard to DnaK/J HSPs, these proteins are widely involved in the plant response to high temperature stress [32], high salt stress [33] and desiccation [34]. According to Scarpeci,HSP 20 was required in a common developmental route in seeds [35], whereas Timperio et al. considered HSP 20 to be a protector against desiccation during seed maturation [36].

Currently, there is no commonly accepted view to explain why different HSPs adopt different abundances, changing profiles during embryo development. Seed dormancy is generally accepted to require limited energy generation and high levels of protein aggregation. hHSPs are ATP-hydrolysis dependent, are larger in size and have more ATP binding sites. Therefore, we suspect that lower ATP concentrations were unfavorable to the functions of hHSPs and DnaK/J HSPs during embryo maturation but were still supportive of HSP 20. Additionally, protein aggregation is closely related to the dehydration status, and proteins with large sizes and hydrophobicity are readily folded due to desiccation. Assuming that dehydration in mature embryos may force the aggregation of HSPs with larger molecular mass but that HSP 20 may be less prone to aggregation was reasonable. These two postulates may explain, at least partially, the proteome results for the embryogenesis-related HSPs.

#### Lipid transfer proteins

**Figure 6:**
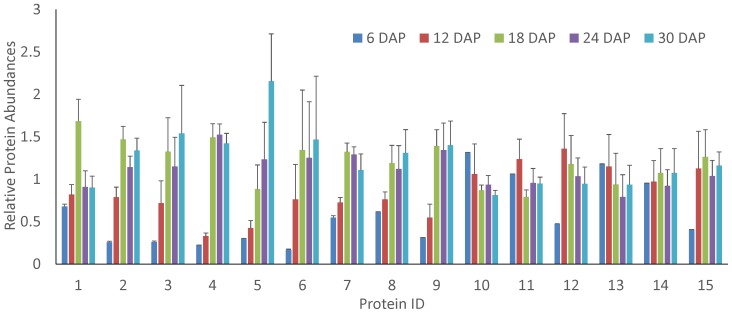
Relative abundance of lipid transfer proteins during embryogenesis. The error bars indicate the standard derivation. 1 indicates the LTP with Locus ID LOC_Os07g11630.1; 2, LOC_Os03g02050.1; 3, LOC_Os12g02320.1; 4, LOC_Os11g40530.1; 5, LOC_Os08g03690.1; 6, LOC_Os05g40010.1; 7, LOC_Os01g60740.2; 8, LOC_Os11g02400.1; 9, LOC_Os10g36170.1.

As a protein family that participates in transporting lipid compounds across the membrane [37], [38], the lipid transfer proteins (LTPs) have been reported to be widely associated with resistance to various plant stresses, including cold [39]–[41], heat [40], dehydration [40], wounding [39], [42], oxidative stress [43], microbial infection [44]–[47] and herbivory [48]. Although the relevant mechanism is largely unknown, the abundant accumulation of LTPs is related to embryogenesis and degradation processes during germination. Sossountzov et al. observed that one LTP was up-regulated during embryogenesis, and Sheoran et al. observed that two LTPs were down-regulated during tomato seed germination [49]. Our data demonstrated for the first time that multiple LTP family members displayed significant changes in response to rice embryogenesis. As illustrated in Figure 6, 90% of the differential LTPs (9/10) had a lower abundance in the early embryogenesis stages, whereas the abundance of many LTPs was stable in the later stages of embryogenesis. Intriguingly, some LTPs appeared to be molecular indicators of embryogenesis. For example, when compared among embryos in all phases of development, LTP24 (LOC_Os08g03690.1) showed the highest abundance in late embryogenesis, whereas LTP163 (LOC_Os07g11630.1) exhibited the highest abundance in the middle stage (6 DAP) of embryogenesis. Thus, the quantitative proteomics analysis of rice embryogenesis will deepen our understanding of LTP function.

#### Proteins related to reactive oxygen species (ROS)

Severe dehydration generally causes membrane leakage and ROS generation [50]. During embryogenesis, many proteins actively regulate ROS to prevent cellular damage. For example, some enzymes related to oxidative stress, such as catalase, ascorbate peroxidase, oxidoreductase, and vacuolar ATPase, were down-regulated, whereas 2-Cys and superoxide dismutase were up-regulated during seed filling in caster, soybean, Arabidopsis and rapeseed [4], [7], [28].

**Figure 7:**
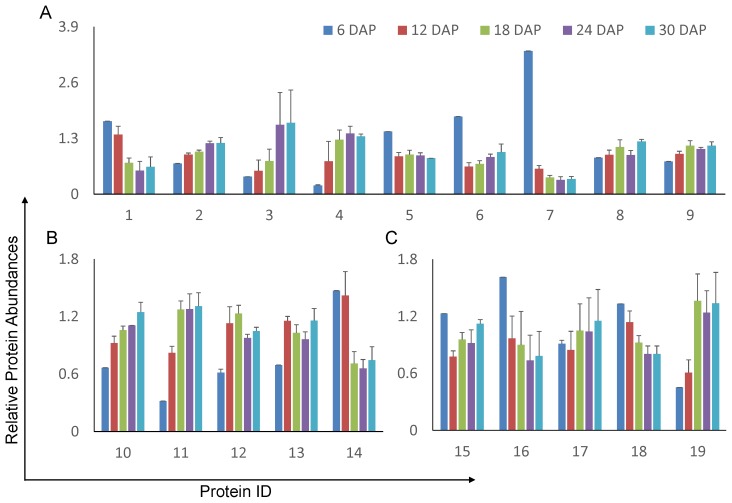
Relative abundance of ROS-related proteins during embryogenesis. (A) Peroxidases. (B) Peroxiredoxins. (C) Doxins. The error bars indicate the standard derivation. 1 indicates the protein with Locus ID LOC_Os04g56180.1; 2, LOC_Os04g59260.1; 3, LOC_Os04g59150.1; 4, LOC_Os06g35520.1; 5, LOC_Os12g07820.1; 6, LOC_Os08g43560.1; 7, LOC_Os03g17690.1; 8, LOC_Os02g44500.1; 9, LOC_Os04g46960.2; 10, LOC_Os05g25850.1; 11, LOC_Os07g44430.1; 12, LOC_Os01g16152.1; 13, LOC_Os06g42000.1; 14, LOC_Os02g09940.1; 15, LOC_Os07g08840.1; 16, LOC_Os03g58130.1; 17, LOC_Os06g21550.1; 18, LOC_Os10g35720.1; 19, LOC_Os04g42930.1.

A total of 16 ROS scavengers were identified in this study, with 14 being embryogenesis-dependent. These proteins were divided into three groups: 9 peroxidases (Figure 7 A), 4 peroxiredoxins (Prx, Figure 7B), and 1 superoxide dismutase (Figure 7 B). Meanwhile, 5 scavenger recovery proteins were found to be embryogenesis-dependent (Figure 7 C). As observed in a previous report [11], three ascorbic peroxidases (LOC_Os12g07820.1, LOC_Os08g43560.1, LOC_Os03g17690.1) were sharply decreased from 6 to 12 DAP and remained at a low concentration in later embryogenesis stages. In addition, our study found 83% of the other peroxidases (5/6) were up-regulated during embryo development. In the four low-efficiency [51] Prx proteins, we not only identified the up-regulation of 1-Cys (LOC_Os07g44430.1), as has been previously reported, but also identified two Prx-II Es (LOC_Os01g16152.1, LOC_Os06g42000.1) and one Prx that were up-regulated during embryogenesis.

During the detoxification of ROS, scavengers must be regenerated by scavenger recovery proteins (thioredoxins or glutaredoxins). The 5 embryogenesis-related scavenger recovery proteins had heterogeneous abundance profiles during rice embryogenesis, indicating that although these proteins are annotated as scavenger recovery proteins, they may play different biochemical roles in the recycling of scavengers. Above all, these proteomic observations deepen our understanding of the molecular mechanism of embryo protection against oxidative stress during embryogenesis and the number of ROS scavengers and scavenger recovery proteins that actively participate in this process.

## Supporting Information

Table S1 Details for identified proteins and peptides.
(XLSX)

Table S2 Spectra information of the three replication.
(XLSX)

Table S3 Quantified proteins groups.
(XLSX)

Table S4 Stress-related proteins in up- and down- regulated protein groups.
(XLSX)

File S1 **Supporting file that contains the five supporting figures.**
(PDF)
