# Supplementary material for: Stress Responsive Proteins Are Actively Regulated during Rice (Oryza sativa) Embryogenesis as Indicated by Quantitative Proteomics Analysis
Source: PLoS One. 2013 Sep 18;8(9):e74229. doi: 10.1371/journal.pone.0074229 (PMC3776822; doi:10.1371/journal.pone.0074229)
Supplement: File S1 — Supporting file that contains the five supporting figures. (PDF) [file pone.0074229.s005.pdf]

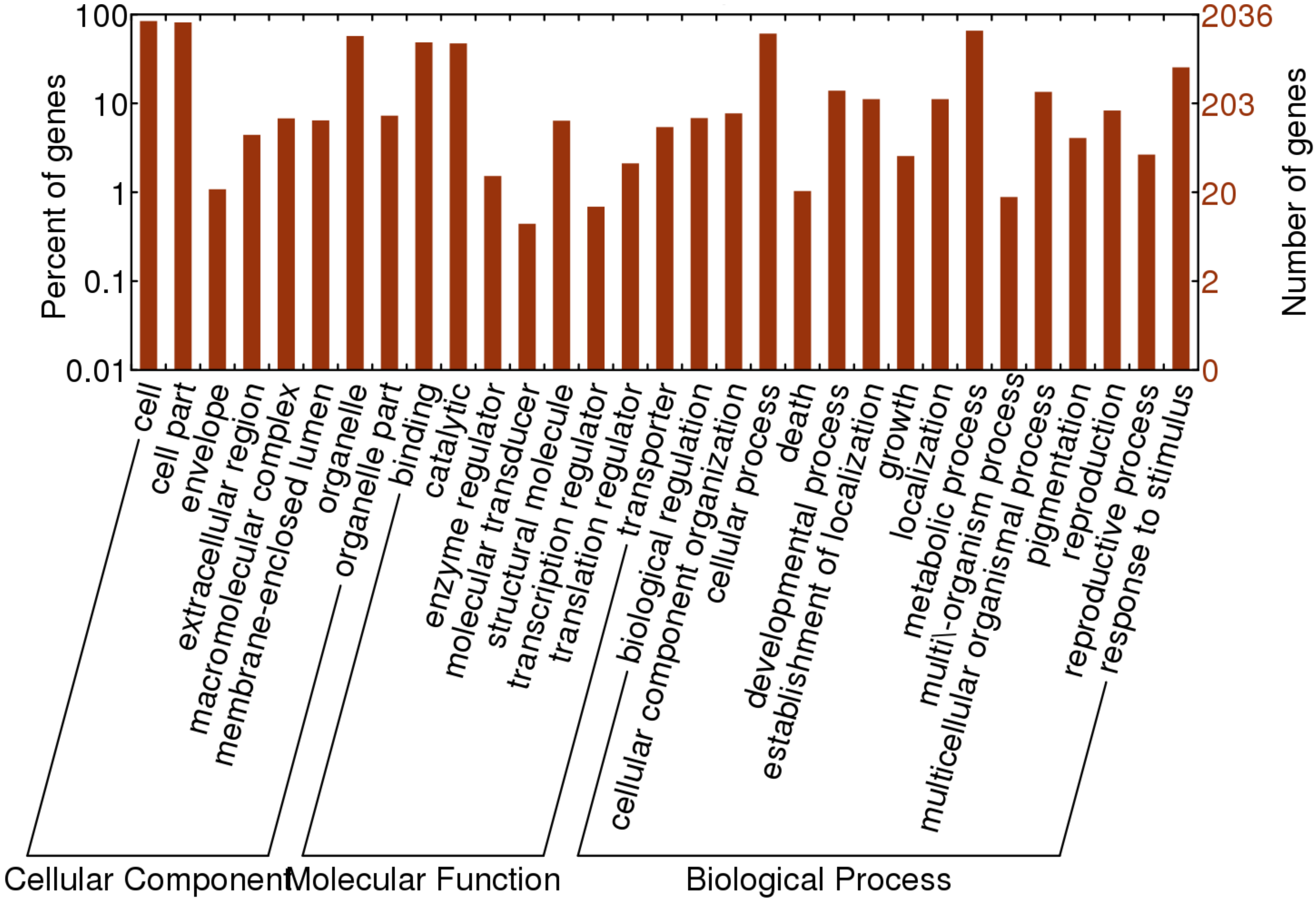

Figure S1 GO classification of identified proteins

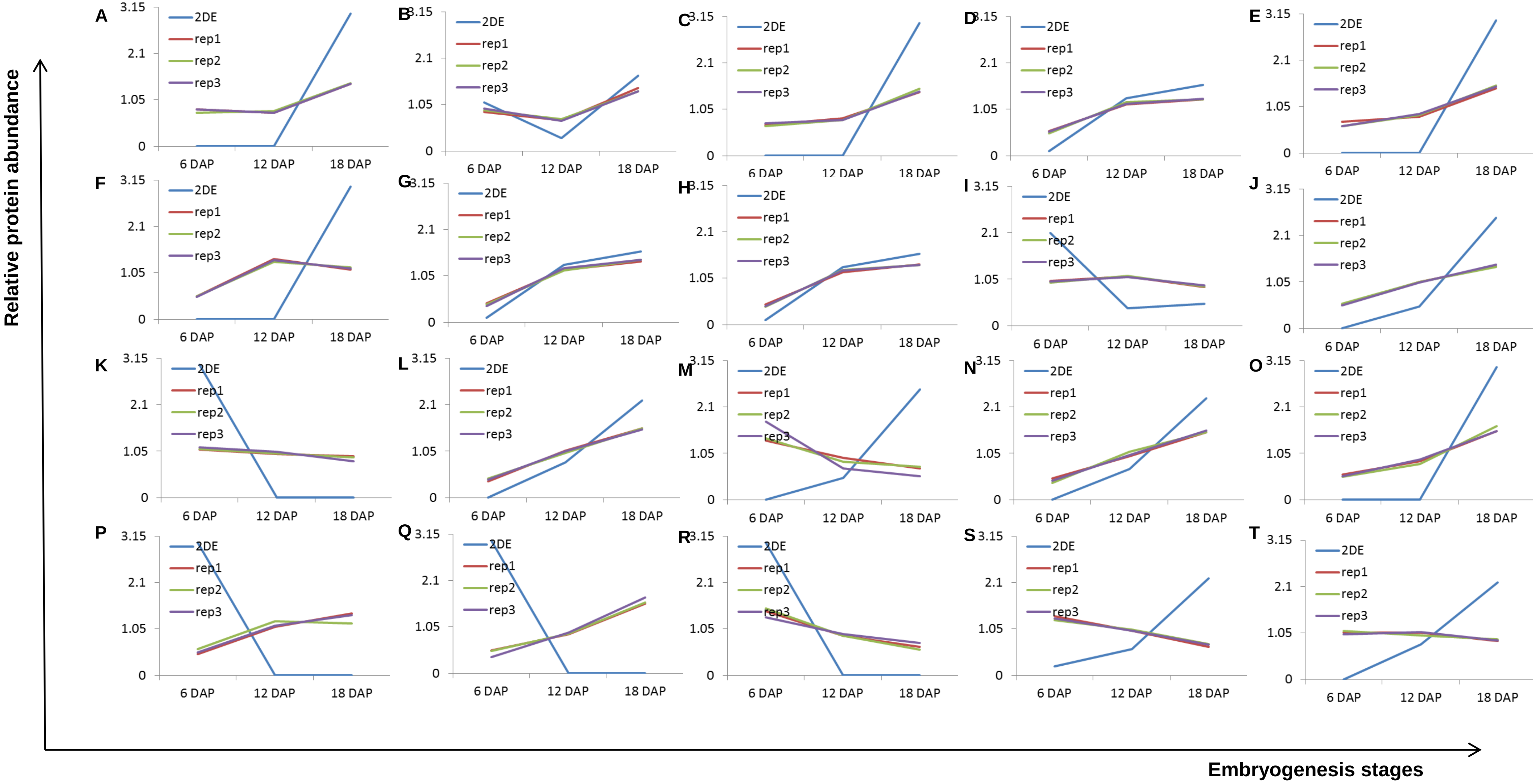

Figure S2 Protein abundances profiles quantified by 2DE and three replicated iTRAQ

From A to T represent the profiles of protein with locus number: LOC_Os03g31360, LOC_Os02g16820, LOC_Os01g55690, LOC_Os03g21790, LOC_Os10g26060, LOC_Os08g03410, LOC_Os03g57960, LOC_Os03g46100, LOC_Os07g47510, LOC_Os07g44430, LOC_Os02g42320, LOC_Os05g04870, LOC_Os07g49400, LOC_Os05g46480, LOC_Os01g50910, LOC_Os12g43140, LOC_Os03g06360, LOC_Os08g02120, LOC_Os05g33380, LOC_Os01g67860,.

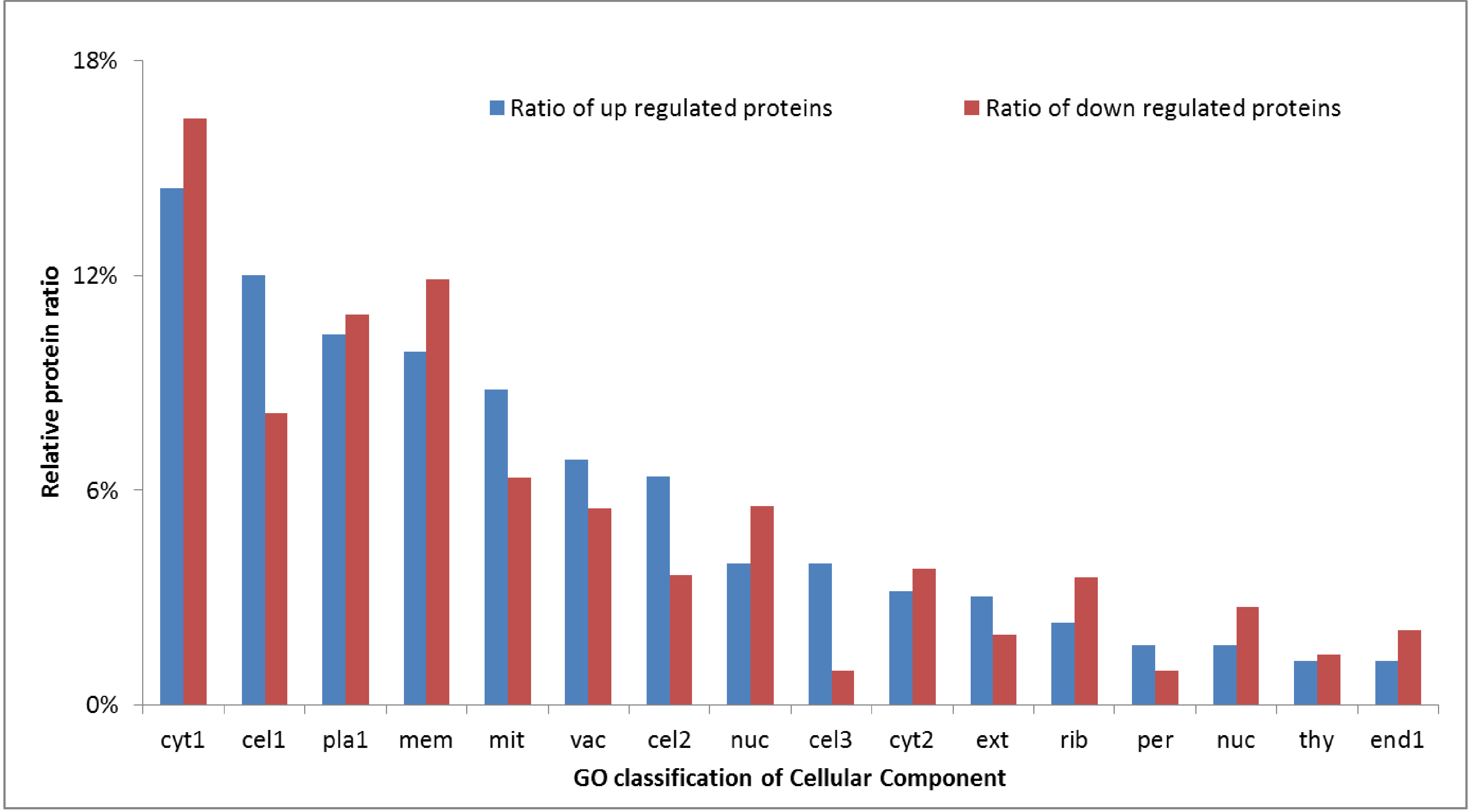

Figure S3 Relative protein ratio in different Cellular Component GO classifications

cyt1 indicate cytosol; cel1 indicate cellular_component; pla1 indicate plastid; mem indicate membrane; mit indicate mitochondrion; vac indicate vacuole; cel2 indicate cell wall; nuc indicate nucleus; cel3 indicate cell; cyt2 indicate cytoplasm; ext indicate extracellular region; rib indicate ribosome; pla2 indicate plasma membrane; per indicate peroxisome; nuc indicate nucleolus; thy indicate thylakoid; end1 indicate endoplasmic reticulum; int indicate intracellular; Gol indicate Golgi apparatus; nuc indicate nucleoplasm; end2 indicate endosome; nuc indicate nuclear envelope.

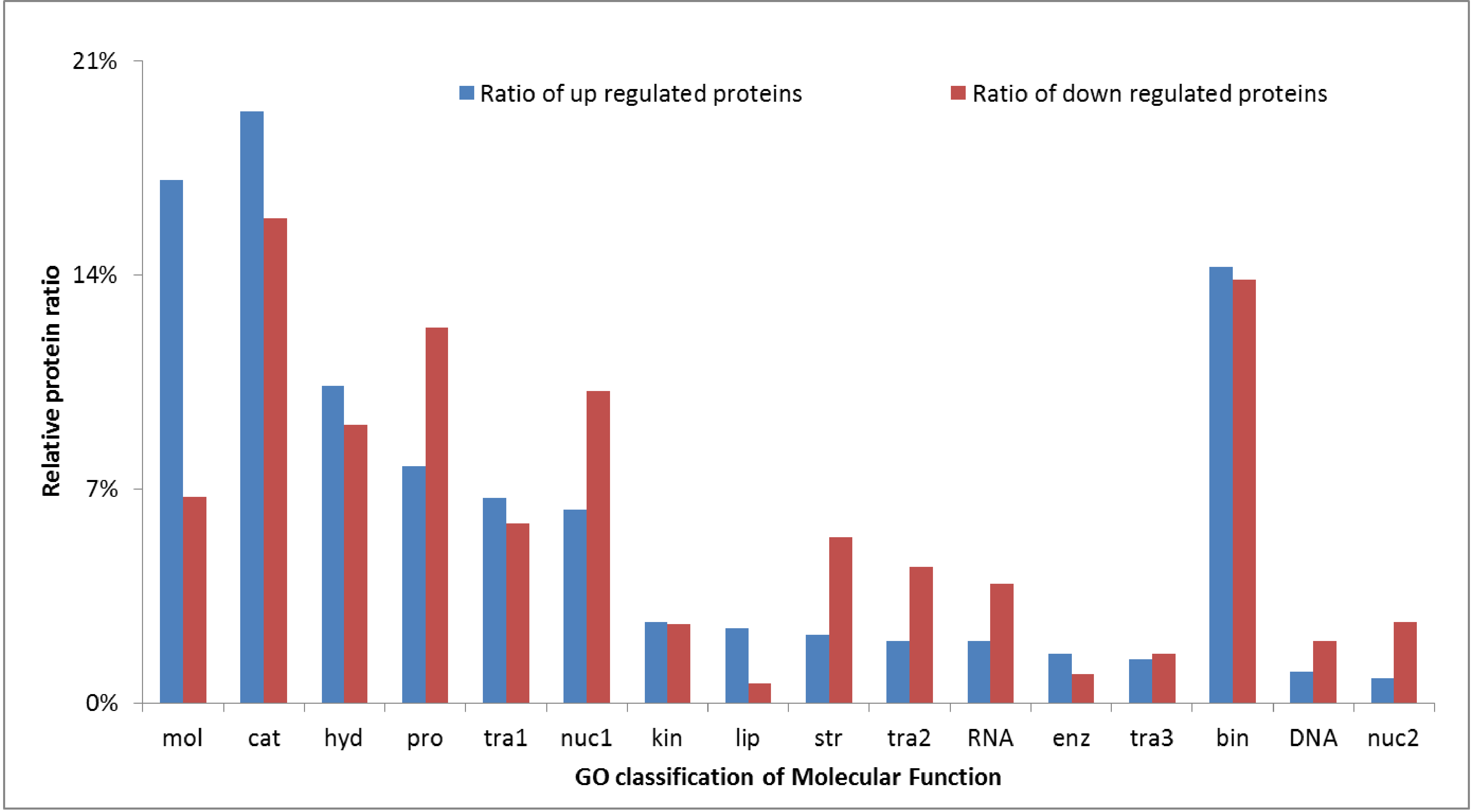

Figure S4 Relative protein ratio in different Molecular Function GO classifications

mol indicate molecular_function; cat indicate catalytic activity; hyd indicate hydrolase activity; pro indicate protein binding; tra1 indicate transferase activity; nuc1 indicate nucleotide binding; kin indicate kinase activity; lip indicate lipid binding; str indicate structural molecule activity; tra2 indicate transporter activity; RNA indicate RNA binding; enz indicate enzyme regulator activity; tra3 indicate translation factor activity, nucleic acid binding; bin indicate binding; DNA indicate DNA binding; nuc2 indicate nucleic acid binding.

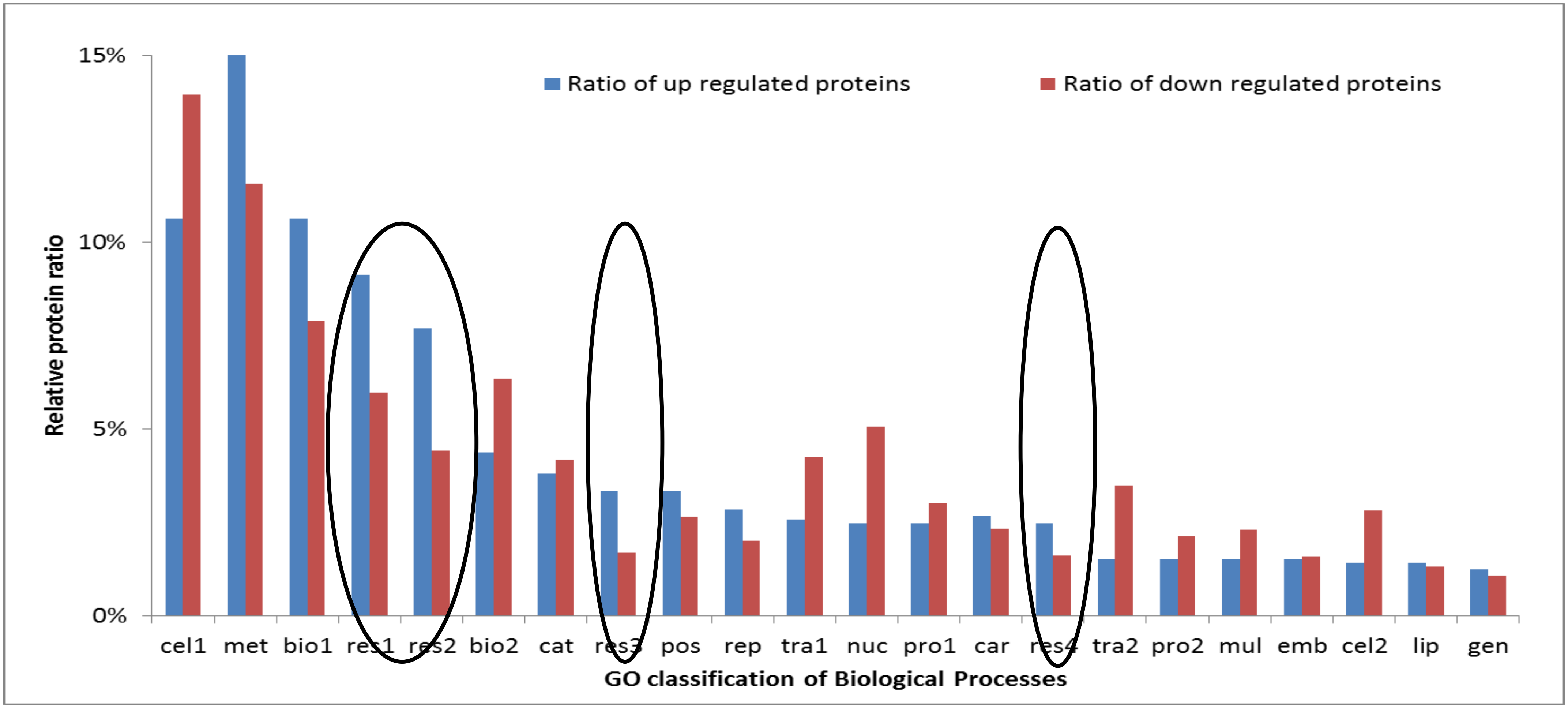

Figure S5 Relative protein ratio in different biological function GO classifications

Three eclipses indicate the GO classification of response to kinds of stimulus.
cel1 indicate cellular process; met indicate metabolic process; bio1 indicate biological_process; res1 indicate response to stress; res2 indicate response to abiotic stimulus; bio2 indicate biosynthetic process; cat indicate catabolic process; res3 indicate response to endogenous stimulus; pos indicate post-embryonic development; rep indicate reproduction; tra1 indicate transport; nuc indicate nucleobase, nucleoside, nucleotide and nucleic acid metabolic process; pro1 indicate protein metabolic process; car indicate carbohydrate metabolic process; res4 indicate response to biotic stimulus; tra2 indicate translation; pro2 indicate protein modification process; mul indicate multicellular organismal development; emb indicate embryo development; cel2 indicate cellular component organization; lip indicate lipid metabolic process; gen indicate generation of precursor metabolites and energy.
